# Temporal Loudness Weights Are Frequency Specific

**DOI:** 10.3389/fpsyg.2021.588571

**Published:** 2021-03-19

**Authors:** Alexander Fischenich, Jan Hots, Jesko Verhey, Daniel Oberfeld

**Affiliations:** ^1^Department of Psychology, Johannes Gutenberg-Universität Mainz, Mainz, Germany; ^2^Department of Experimental Audiology, Otto von Guericke University Magdeburg, Magdeburg, Germany

**Keywords:** loudness, frequency specific, intensity discrimination, temporal weights, auditory

## Abstract

Previous work showed that the beginning of a sound is more important for the perception of loudness than later parts. When a short silent gap of sufficient duration is inserted into a sound, this primacy effect reoccurs in the second sound part after the gap. The present study investigates whether this temporal weighting occurs independently for different frequency bands. Sounds consisting of two bandpass noises were presented in four different conditions: (1) a simultaneous gap in both bands, (2) a gap in only the lower frequency band, (3) a gap in only the higher frequency band, or (4) no gap. In all conditions, the temporal loudness weights showed a primacy effect at sound onset. For the frequency bands without a gap, the temporal weights decreased gradually across time, regardless of whether the other frequency band did or did not contain a gap. When a frequency band contained a gap, the weight at the onset of this band after the gap was increased. This reoccurrence of the primacy effect following the gap was again largely independent of whether or not the other band contained a gap. Thus, the results indicate that the temporal loudness weights are frequency specific.

## Introduction

In loudness judgments of time-varying sounds, higher perceptual weights are assigned to the first few hundred milliseconds of a sound compared to later temporal portions (e.g., Namba et al., 1976; Ellermeier and Schrödl, 2000; Plank, 2005; Pedersen and Ellermeier, 2008; Dittrich and Oberfeld, 2009; Rennies and Verhey, 2009; Ponsot et al., 2013). This primacy effect can be described by an exponential decay function with a time constant of about 300 ms (Hots et al., 2018; Oberfeld et al., 2018; Fischenich et al., 2019). The temporal weighting was reported to be largely independent of the spectral weighting (Oberfeld et al., 2012). Pedersen and Ellermeier (2008) showed that when the spectrum changes abruptly within a contiguous sound, a second primacy effect is observed on the second sound part. In a recent study, Fischenich et al. (2020) reported that such a reoccurrence of the primacy effect is also obtained when a silent gap is inserted into the sound. Their data showed that after a gap of at least 350 ms, a significant primacy effect reoccurred on the second sound part. The initial primacy effect on the first temporal segments of the sound was reduced, and at the onset of the sound after the silent gap, the weights on the first two to three segments (segment duration 100 ms) following the gap were increased relative to the weights assigned to the subsequent segments. This primacy effect on the second sound part became more pronounced when the gap duration was further increased.

In the present study, we investigated whether the effects of a silent gap inserted into a sound on the temporal loudness weights occur specifically for each presented frequency band, or if a gap in one of the frequency bands affects the temporal weights for the entire sound. Put differently, are temporal loudness weights frequency specific? The answer to this question is, among others, important for modeling temporal loudness weights. In a model, one could apply temporal weights independently for each auditory filter (i.e., before spectral integration of loudness), or to the sound as a whole (i.e., after spectral integration). How temporal weights are assigned is also important for the understanding of the everyday sound perception. In our acoustic environment, in many situations background sounds produced by other people, animals, technical devices, or weather phenomena are present most of the time. In this context, it is an interesting question whether different spectral components interact with each other in terms of the temporal loudness weights when the overall loudness of a time-varying sound is judged.

In most previous experiments on temporal loudness weights, a broadband noise (Pedersen and Ellermeier, 2008; Dittrich and Oberfeld, 2009; Oberfeld et al., 2018), a single narrowband noise band (Rennies and Verhey, 2009; Fischenich et al., 2019) or a pure tone (Ponsot et al., 2013) was presented. The corresponding data thus do not provide an answer to the question of whether the temporal weights are assigned on a frequency-specific basis.

Studies on temporal loudness weights (Oberfeld and Plank, 2011; Oberfeld, 2015; Fischenich et al., 2019) discussed the possibility that the temporal weighting pattern observed for loudness judgments of time varying sounds is caused by the response characteristics of auditory nerve (AN) fibers. AN fibers show an initial peak in their firing rate at the onset of a sound (Kiang et al., 1965). With a preceding masker a pronounced peak also occurs at the onset of a sound after a certain silent interval. The necessary duration of that silent interval for a pronounced peak to occur varies between different fibers (e.g., Rhode and Smith, 1985; Relkin and Doucet, 1991). As the inner hair cells that innervate the AN fibers are frequency specific, the recovery of the firing rate is also frequency specific (e.g., Harris and Dallos, 1979) and thus would support the assumption of frequency-specific temporal weights.

Another potential source of the temporal weighting patterns, which also predicts frequency-specific temporal weights, are forward-masking effects on the intensity resolution (e.g., Zeng et al., 1991). Such forward-masking effects might result in higher intensity resolution for the first few temporal segments of a longer sound compared to later segments. In a loudness judgment task, this could induce a strategy of attending primarily to the beginning of the sound where the intensity resolution is higher (for an in-depth discussion see Fischenich et al., 2020). Because Zeng and Turner (1992) found that maskers with frequency components two to three octaves away from the signal frequency did not affect the intensity resolution for the signal, this explanation for the primacy effect would also predict frequency-specific weights.

In contrast, a potential argument for an *interaction* of the temporal weights across frequency is that the bands may suppress each other. Two-tone suppression is observed over large spectral distances (e.g., Houtgast, 1974; Ernst et al., 2010). During a silent gap in one of the bands, the other band is no longer suppressed, and thus the auditory fibers encoding this frequency range might be more strongly activated during the gap in the other band compared to those positions in time where both bands are presented simultaneously. In perceptual terms, the loudness of a given frequency band could be reduced by suppression caused by a simultaneously presented band. In such a case, the loudness of the ongoing band should be higher during the gap in the other band. A phenomenon that could cause a change in the temporal weighting patterns in such a situation is *loudness dominance* (Berg, 1990), which has been shown in several studies on temporal loudness weights (e.g., Lutfi and Jesteadt, 2006; Oberfeld, 2008a, 2015; Ponsot et al., 2013). Loudness dominance describes the effect that temporal portions of a sound that are, on average, higher or lower in level or loudness compared to the rest of the sound receive higher or lower weights, respectively. If the effect of loudness dominance precedes spectral loudness summation, then the release from suppression during the gap in the other band might render the segments of the band that contains no gap presented during the gap in the other band *louder* than the segments presented simultaneously with the other band. In this case, higher weights on the temporal segments of the band that does not contain a gap can be expected during the gap in the other band.

In contrast, if loudness dominance takes place *after* spectral loudness summation, one may expect the opposite pattern. During the gap in one of the bands, the *overall* loudness of the sound (across frequencies) is *reduced*. Thus, the loudness dominance effect would cause the weights for the temporal segments of the band that did not contain a gap to be *reduced* during the duration of the gap in the other band. After the gap, when both bands are presented again, the weights on the band that contained no gap should increase again because the overall loudness of the sound increases.

To answer the question of whether temporal loudness weights are frequency specific or not, the present study used stimuli consisting of two frequency bands that were separated by more than two critical bands in order to minimize simultaneous masking. The two frequency bands were presented in four conditions. Either none of the bands, only the lower band, only the higher band, or both bands contained a silent gap in the temporal center of the sound. Using a behavioral reverse-correlation approach, temporal perceptual weights were measured for each of the two frequency bands. To this end, independent level variations were imposed on temporal segments in the two bands (Oberfeld et al., 2012). The rationale was that if the temporal weights are frequency specific, then the temporal weights on a given frequency band should not be affected by the presence or absence of a temporal gap in the other frequency band.

The study was organized into two experiments. Experiment 1 presented a gap duration of 500 ms. Experiment 2 was conducted to replicate the findings of Experiment 1 in an independent group of participants. Also, we presented slightly longer gap duration of 700 ms compared to the 500 ms in Experiment 1. Two gap durations were used to assess potential differences in the pattern of the weights due to the gap duration. Such differences were observed in previous work on temporal loudness weights of sounds including a temporal gap (Fischenich et al., 2020).

## Experiment 1

### Method

#### Listeners

Eight normal hearing listeners (four female, four male, age 18–29 years) participated in this experiment. Their hearing thresholds were measured by Békésy audiometry with pulsed 270-ms pure tones and were lower than or equal to 15 dB HL on both ears in the frequency range between 125 Hz and 8 kHz. All participants were students from the Johannes Gutenberg-Universität Mainz and received partial course credit for their participation. The experiment was conducted according to the principles expressed in the Declaration of Helsinki. All listeners participated voluntarily and provided informed written consent, after the topic of the study and potential risks had been explained to them. They were uninformed about the experimental hypotheses. The Ethics Committee of the Institute of Psychology of the Johannes Gutenberg-Universität Mainz approved the study (reference number 2016-JGU-psychEK-002).

#### Stimuli and Apparatus

The stimuli consisted of two level-fluctuating noise bands, each comprising 10 or 15 bandpass-filtered temporal noise segments. The total number of segments depended on the condition, as outlined below. To reduce the intrinsic envelope fluctuations of the noise within a segment, low-noise noise was used (Hartmann and Pumplin, 1988). The present study generated low-noise noise using the first method of Kohlrausch et al. (1997) with two iterations. To generate low-noise noise, first, a Gaussian white noise was generated and filtered with a fast Fourier transform (FFT) based bandpass filter. The amplitudes of all frequency components outside the desired frequency range were set to zero. The cutoff frequencies were 200 Hz (2 Bark) and 510 Hz (5 Bark) for the lower noise band (referred to as LB), and 3150 Hz (16 Bark) and 5300 Hz (19 Bark) for the higher noise band (referred to as HB). Second, the following steps were iterated two times: (i) The Hilbert envelope was calculated, (ii) the stimulus was divided by its Hilbert envelope, and (iii) it was filtered using the FFT-based bandpass filtering, as described above. For each temporal segment, a new random Gaussian noise was generated, and the signal processing steps described above were applied to it. Each noise segment had a duration of 120 ms including 20-ms cos^2^ ramps at segment on- and offset. Contiguous segments were presented, with a temporal overlap of 20 ms. Random level fluctuations were created by assigning a sound pressure level drawn independently and at random from a normal distribution to each temporal segment on each trial (see section “Procedure” for details).

All sounds were generated digitally with a sampling frequency of 44.1 kHz and a resolution of 24 bit, D/A-converted by an RME ADI/S, attenuated by a TDT PA5 programmable attenuator, buffered by a TDT HB7 headphone buffer, and presented diotically via Sennheiser HDA 200 circumaural headphones. The reproducing system was calibrated according to IEC 318 (1970), and free-field equalized as specified in ISO 389-8 (2017). Participants were tested in a double-walled sound-insulated chamber. Instructions were presented on a computer screen.

#### Experimental Conditions

**FIGURE 1:**
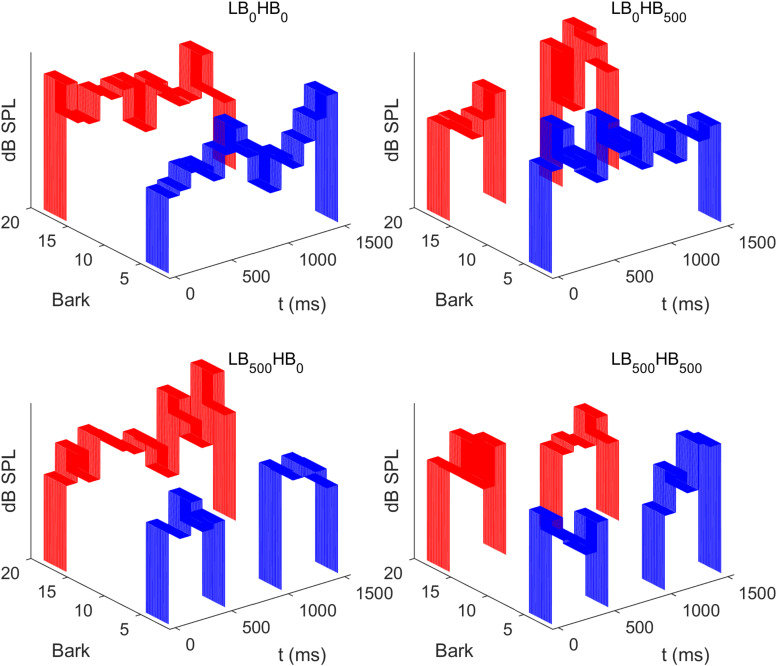
Schematic spectrograms of the level fluctuating sounds presented in the four different conditions of Experiment 1. Each sound comprised two frequency bands (blue: lower band, LB, 2–5 Bark; red: higher band, HB, 16–19 Bark). Independent random level fluctuations were imposed on each of the 100-ms segments. In the example displayed here the same distribution mean was used for the higher and the lower band. In contrast, in the experiment, these means differed between the two bands as the HB was loudness matched to the LB (see section “Procedure”). Depending on the condition, a 500-ms silent gap was inserted in none of the frequency bands (LB_0_HB_0_), in only the higher band (LB_0_HB_500_), in only the lower band (LB_500_HB_0_), or in both bands (LB_500_HB_500_).

The two noise bands were presented simultaneously in four different conditions, which are displayed in Figure 1. In the first condition, each of the two noise bands consisted of 15 contiguous segments. This condition is referred to as LB_0_HB_0_, where 0 indicates a gap duration of 0 ms (no gap). In the second condition, referred to as condition LB_500_HB_0_, a gap of 500 ms was inserted between segments 5 and 6 of LB, while no gap was presented in HB, so that the latter noise band contained 15 contiguous temporal segments. In the third condition (LB_0_HB_500_), a gap was inserted in the middle of HB whereas LB did not contain a gap. Finally, in the fourth condition (LB_500_HB_500_), both LB and HB were presented with a gap of 500 ms duration.

#### Procedure

To estimate temporal loudness weights, we used an established experimental paradigm from previous experiments (e.g., Pedersen and Ellermeier, 2008; Oberfeld and Plank, 2011). On each trial, the two noise bands were presented. Depending on the experimental condition (see Figure 1), each noise band consisted of either 10 or 15 100-ms segments. For each trial, the segment levels of both bands were drawn independently and at random from a truncated normal distribution. With equal probability and uniformly for both bands, either a level distribution with a lower mean or a distribution with a higher mean was selected on each trial. The main aim of the introduction of two different mean levels was to adjust the difficulty of the task and to motivate the listeners by giving feedback about the “correctness” of their response. The level difference between the two distribution means was selected so that the listeners were able to respond with roughly 70% correct.

For LB, the level distribution with higher mean had a mean level of *μ*_*H*_*low*_ = 52.75 dB SPL and the distribution with lower mean had a mean level of *μ*_*L*_*low*_ = 51.25 dB SPL. In an initial session, HB was loudness-matched to LB for each listener in an adaptive two-interval forced-choice procedure (see Supplementary Material “Loudness matching” for details of the matching procedure). This was done to eliminate the effect of “loudness dominance,” i.e., the effect that stimulus components with on average higher loudness receive higher weights (e.g., Berg, 1990; Oberfeld, 2008c; Oberfeld and Plank, 2011; Oberfeld et al., 2013). Averaged across the eight listeners, the level difference between HB and LB at equal loudness was −0.27 dB (*SD* = 4.11 dB) and the resulting mean sound pressure levels of HB were *μ*_*H_high*_ = 52.48 dB SPL and *μ*_*L_high*_ = 50.98 dB SPL. The individual sound pressure level differences between HB and LB are displayed in Supplementary Table 1. In the final session, loudness matches were obtained again for each listener, to assess if the matches remained stable across time. The test–retest reliability was high, indicating adequate stability across time (see Supplementary Material “Loudness matching” for information on stability).

The standard deviation of all level distributions was *σ* = 2.5 dB. Overly loud or soft segments were avoided by limiting the range of possible sound pressure levels to *μ* ± 3 ⋅ *σ*. On each trial, participants judged the overall loudness (i.e., the loudness of both frequency bands across the entire stimulus duration, encompassing potential silent temporal gaps) by deciding whether the presented sound had been louder or softer in comparison to previous trials within the same experimental block. Thus, a one-interval, *absolute identification task* (Braida and Durlach, 1972) with a virtual standard (e.g., Nachmias, 2006) was used.

The minimum silent interval between trials was 1500 ms. The next trial never started before the response to the preceding trial had been given. Trial-by-trial feedback was provided during the first seven trials of each block so that the participants could easily adopt a decision criterion for the new experimental condition. Those trials were not considered for the data analysis. A summarizing feedback was provided each time 50 trials were completed. It contained the number of correct and false answers, percent correct and the number of *μ*_*H*_ and *μ*_*L*_ trials as well as the number of “louder” and “softer” responses. Note that a response was classified as “correct” if the response (“louder”/”softer”) matched the mean of the distribution that the stimulus’ segment levels were drawn from (*μ*_*H*_/*μ*_*L*_).

To obtain a sufficient number of observations for the weight estimation, we presented 80 trials per temporal segment (cf. Oberfeld et al., 2018). As there were four different conditions in which the number of the temporal segments varied between a total of 20 (condition LB_500_HB_500_), 25 (conditions LB_500_HB_0_ and LB_0_HB_500_) and 30 segments (condition LB_0_HB_0_), we presented 1600, 2000, and 2400 trials per condition, amounting to a total of 8000 trials per listener.

#### Sessions

Each listener participated in nine experimental sessions, each containing 1000 trials of the loudness judgment task (300 for condition LB_0_HB_0_, 250 for LB_500_HB_0_, 250 for LB_0_HB_500_, and 200 for LB_500_HB_500_). Additionally, there was an initial session in which audiometric thresholds were measured, loudness matches were obtained, and practice blocks of the loudness judgment task were presented for all of the four conditions. The practice blocks were excluded from the data analysis. Within each session, sounds of the same condition were arranged into blocks with the above mentioned trial numbers. The order of conditions was chosen randomly. At the end of the final session, a second set of loudness matches (i.e., loudness matching of HB to LB; see Supplementary Material “Loudness matching” for details) was obtained from each listener. The duration of each session was approximately 60 min, including a mandatory pause of about 5 min.

#### Data Analysis

The perceptual weights representing the importance of the 10–15 temporal segments of both bands for the decision in the loudness judgment task were estimated from the trial-by-trial data via multiple logistic regression. The decision model assumed that the listener compares a weighted sum of the segment levels of both bands to a fixed decision criterion, and responds that the sound was of the “louder” type if the weighted sum exceeds the criterion (a detailed description of the decision model is provided by Oberfeld and Plank, 2011). If the weighted sum is smaller than the criterion, then it is assumed that the listener classifies the sound as “softer.” In the data analysis, the binary responses (”louder” or “softer”) served as the dependent variable. The predictors (i.e., the 20, 25, or 30 segment levels) were entered simultaneously. The regression coefficients were taken as the decision weight estimates. Because the segment levels were drawn independently for each frequency band, this allowed for the detection of possible interactions between the bands in the observed temporal weights, especially in situations in which one band did contain a gap while the other one was contiguous.

A separate logistic regression model was fitted for each combination of listener and condition. The model included an intercept term so that potential biases toward one of the two responses were accounted for. The percentages of “softer” and “louder” responses as well as the SDT decision criterion *c* and the sensitivity in terms of *d′* is shown in Table 1 for each listener in Experiment 1. A value of *c* = 0 represents unbiased responses. In general, the responses of the participants did not show strong response biases. As stated in the Methods section, we presented seven trials with trial-by-trial feedback at the beginning of each experimental block, so that the participants could easily adopt a decision criterion for the new experimental condition. Those trials were not considered for the data analysis. We assume that the decision criterion remained relatively stable across the remaining trials of the block. Still, it is of course possible that the listeners used information from preceding trials when forming their decision (Stewart et al., 2005), resulting in potential small shifts in the response criterion from trial to trial. Such a variability in the response criterion would reduce the goodness of fit of the logistic regression models that assumed a fixed response criterion. However, since the *relative* contributions of the different segments to the decision were of interest, rather than the absolute magnitude of the regression coefficients, this was of no significance for the research question of the present paper.

**TABLE 1:** Average percentages of “softer” and “louder” responses as well as the SDT decision criterion *c* and the sensitivity in terms of *d*′ for each listener in Experiment 1.

Listener	% “louder”	% “softer”	Mean of c	SD of *c*	Mean of *d*′	SD of *d*′
1	0.54	0.46	–0.12	0.26	1.00	0.20
2	0.44	0.56	0.18	0.16	0.84	0.28
3	0.56	0.44	–0.17	0.23	0.77	0.27
4	0.47	0.53	0.10	0.23	1.15	0.22
5	0.59	0.41	–0.25	0.25	0.95	0.28
6	0.48	0.52	0.04	0.21	1.12	0.21
7	0.48	0.52	0.07	0.19	1.45	0.17
8	0.50	0.50	–0.01	0.13	1.27	0.18

To focus the analyses on the *relative* contributions of the different segments to the decision, the regression coefficients for each frequency band were normalized so that the mean of the absolute values of the first five and the final five segments was 1.0. Thus, for each frequency band, exactly 10 segments contributed to the computation of the normalization factor in both the conditions with and without a gap, and the five middle weights in the conditions without a gap were not included in the normalization. This was done in order to avoid that the additional five middle segments presented in conditions without a gap lead to a different scaling of the weights compared to the conditions with a gap. The normalization per frequency band was done to compare the weights assigned to a specific band between the different conditions, independent of the weights assigned to the other band. We also conducted all analyses reported within this study for a normalization of the weights based on the mean of the absolute values of the first five segments for each frequency band. This kind of normalization was suggested by a reviewer and led to almost the same pattern of results as the normalization which was used within this study (see Supplementary Material “alternative normalization” for detailed plots and analyses).

Due to the sampling of all segment levels from either the distribution with higher or the distribution with lower mean, the segments levels were weakly correlated. Across all experiments reported in this paper, the maximum pairwise Pearson correlation between two segments levels was *r* = 0.12 (average *r* across listeners = 0.08). Multiple logistic regression analyses do not require the predictors to be uncorrelated. According to the Gauss–Markov theorem (Gauss, 1821), the estimated regression parameters from a (generalized) linear model are still unbiased when the predictors are correlated. We checked the validity of this assumption by fitting separate multiple logistic regression models to trials with segment levels sampled from the distribution with higher or the distribution with lower mean, for each combination of listener and condition. The averages of the normalized segment weights across the two level distributions per listener and condition were virtually identical (adjusted *R*^2^ ≥ 0.975) to the normalized weights estimated by fitting the logistic models to the trials from both level distributions simultaneously.

A summary measure of the predictive power of a logistic regression model is the area under the Receiver Operating Characteristic (ROC) curve (for details see Dittrich and Oberfeld, 2009). Areas of 0.5 and 1.0 correspond to chance performance and perfect performance of the model, respectively. Across the 32 (eight listeners, four conditions) fitted logistic regression models, the area under the ROC curve ranged between 0.70 and 0.88 (*M* = 0.80, *SD* = 0.05), indicating on average reasonably good predictive power (Hosmer and Lemeshow, 2000).

The individual normalized temporal weights were analyzed with repeated-measures analyses of variance (rmANOVAs) using a univariate approach with Huynh–Feldt correction for the degrees of freedom (Huynh and Feldt, 1976). The correction factor $\tilde{\varepsilon }$ is reported, and partial η^2^ is reported as measure of association strength. An α-level of 0.05 was used for all analyses. If not stated otherwise, calculations were done with R 3.6.1 and R Studio 1.2.1335.

### Results

The average sensitivity in terms of *d*′ is shown in Table 2 for each of the four conditions. There was a significant effect of condition on *d*′, *F*(3,21) = 5.19, $\tilde{\varepsilon }$ = 0.701, *p* = 0.019, $\eta_{p}^{2}$ = 0.426, with slightly higher mean sensitivity when both bands contained a gap (LB_500_HB_500_), and slightly lower sensitivity when none of the bands contained a gap (LB_0_HB_0_).

**TABLE 2:** Mean sensitivity (*d*′) in the four different conditions of Experiment 1.

Condition	Mean of *d*′	SD of *d*′
LB_0_HB_0_	0.99	0.25
LB_0_HB_500_	1.09	0.25
LB_500_HB_0_	1.08	0.21
LB_500_HB_500_	1.17	0.13

**N* = 8.*

**FIGURE 2:**
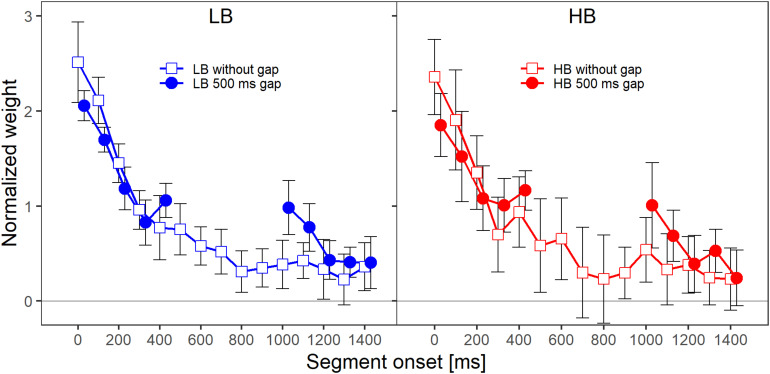
Mean normalized weights as a function of segment onset for Experiment 1, averaged across spectral context. The two panels show the weights for the two frequency bands, lower band (LB, **left panel**) and higher band (HB, **right panel**). The frequency band is also indicated by the colors that were introduced in Figure 1 (LB: blue, HB: red). The different symbols and separate lines within each panel indicate whether the band did or did not contain a gap (open squares: without gap, filled circles: 500-ms gap). Error bars show 95% confidence intervals (CIs). Note that for better visibility, the two lines are shifted slightly against each other along the *x*-axis.

Figure 2 shows the mean normalized temporal weights assigned to the two frequency bands. Filled circles and open squares represent conditions where the plotted band did or did not contain a gap, respectively. For each of the plotted lines, the weights are averaged across the spectral context, that is, across the two conditions where the other frequency band either did or did not contain a gap. For both frequency bands, the patterns of the mean weights in both conditions (with and without a gap) showed a clear primacy effect at the beginning of the sound, in the sense that the weight on the first segment was higher than the weights on the following segments.

When a band contained a gap, the weight assigned to the first segment after the gap was higher compared to the condition in which the band did not contain a gap. Note that, in addition to this reoccurrence of the primacy effect after the silent gap, the primacy effect at the beginning of the sound was reduced when the band contained a silent gap. To investigate whether descriptive differences in the patterns of temporal weights can be explained by the stimulus properties in this condition (e.g., the frequency band that is concerned, whether the band contains a gap, or whether the other band contains a gap) one always has to compare the temporal weights for a given condition to a suitable control condition (e.g., HB without a gap vs. HB with a gap). This is necessary because, for example, even without a gap a difference between the segments weights is expected for the segments following the gap region as the weights tend to decline as a function of segment number/temporal onset even for later segments. The normalized temporal weights were analyzed with an rmANOVA with the within-subjects factors segment number (1–10 when the band contained a gap, 1–5 and 11–15 when the band did not contain a gap), target frequency band (LB, HB), target gap (no gap, 500-ms gap), and context (other band with 500-ms gap, other band without a gap). The rmANOVA showed a significant main effect of segment number, *F*(9,63) = 40.06, $\tilde{\varepsilon }$ = 0.434, *p* < 0.001, $\eta_{p}^{2}$ = 0.851, highlighting the non-uniform temporal weighting patterns. The target gap × segment number interaction was also significant, *F*(9,63) = 5.24, $\tilde{\varepsilon }$ = 0.933, *p* < 0.001, $\eta_{p}^{2}$ = 0.429, indicating that the pattern of the weights of the segments differed depending on whether a band was presented with or without a gap. This supports the observation that for the bands that contained a gap (filled circles in Figure 2), the weights assigned to the first segments following the gap were higher than the weights assigned to the same temporal positions when a band did not contain a gap (open squares in both panels of Figure 2). Thus, as expected, we observed a significant reoccurrence of the primacy effect.

**FIGURE 3:**
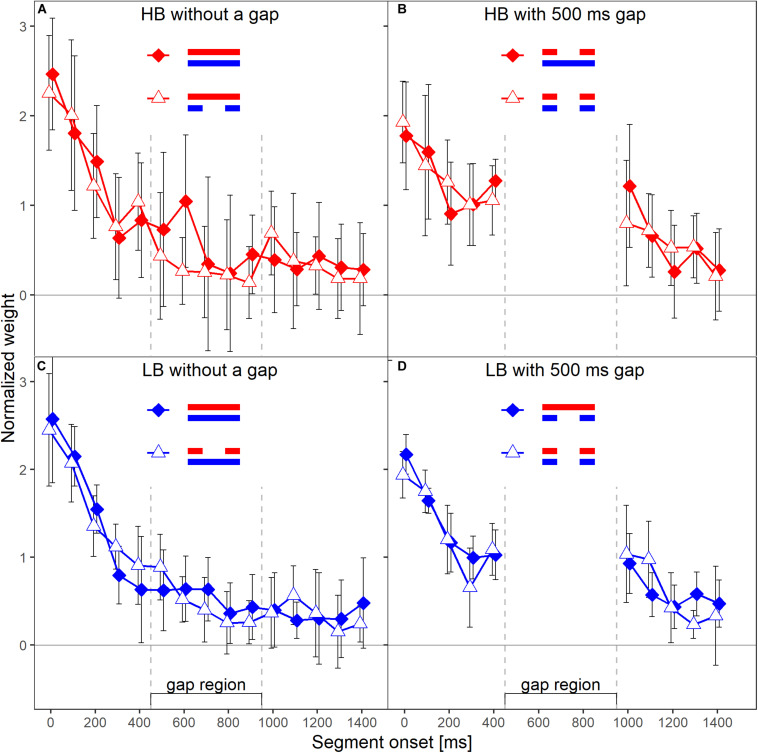
Mean normalized temporal weights as a function of segment onset for Experiment 1. Upper panels **(A,B)** show the weights for the HB, lower panels **(C,D)** show the weights for the LB. The frequency band is also indicated by color, red = HB, blue = LB. Panels in the left column show the weights in the conditions without a gap in the analyzed band, panels on the right show the weights in the conditions with a gap. In each panel, the two different lines indicate the two different context conditions. Solid diamonds show the weights in the conditions in which the other band did not contain a gap, open triangles show the weights in the conditions in which the other band contained a gap. Error bars show 95% confidence intervals (CIs). Note that for better visibility, the two lines are shifted slightly against each other along the *x*-axis.

The primary aim of our experiment was to test whether the temporal weights for a given frequency band are unaffected by the presence or absence of a gap in the other frequency band, and thus are frequency specific. Each panel in Figure 3 shows the normalized weights for one band and depending on whether or not the plotted band did or did not contain a silent gap. The two lines shown in each panel represent the two spectral context conditions, that is, the presence or absence of a gap in the other frequency band. To answer the question whether the weights in one band are affected by the presence (or absence) of a gap in the other band, one has to compare the two lines in each panel of Figure 3. For example, the filled symbols in the left upper panel represent the weights observed for the higher band without gap, in the condition where the lower band also did not contain a gap (LB_0_HB_0_; see Figure 1). The open symbols represent the weights observed for the HB without gap, but this time in the condition where the LB contained a 500-ms gap (LB_500_HB_0_). Except for the segment with onset at 600 ms after sound onset, the weights in the two conditions were very similar. Thus, the temporal weights assigned to the higher band without gap were hardly affected by the temporal structure (with or without gap) of the other band. The same trend can be observed for the lower band without gap (left lower panel), for the lower band with gap (right lower panel), and, to a limited extent, also for the higher band with gap (right upper panel).

**FIGURE 4:**
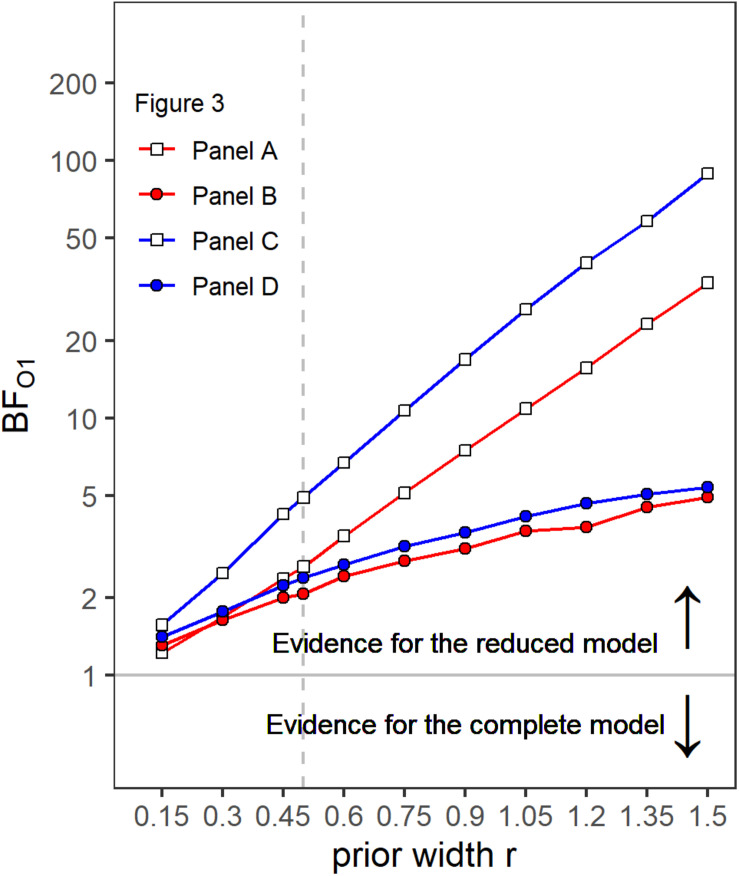
Bayes factors (BF_01_) as a function of prior width for the four different panels of Figure 3. Values of BF_01_ greater than 1.0 indicate a higher posterior probability for the reduced model not containing a segment × context interaction, compared to the complete model including a segment × context interaction.

A first indicator that the temporal weights were frequency specific is that in the rmANOVA reported above, there were no significant interactions of the factor context (presence or absence of a gap in the other band) with segment number [*F*(9,63) = 1.15, $\tilde{\varepsilon }$ = 0.945, *p* = 0.347, $\eta_{p}^{2}$ = 0.141], segment number and target gap [*F*(9,63) = 1.58, $\tilde{\varepsilon }$ = 0.952, *p* = 0.144, $\eta_{p}^{2}$ = 0.185], or segment number, target gap and target frequency band [*F*(9,63) = 1.22, $\tilde{\varepsilon }$ = 1, *p* = 0.297, $\eta_{p}^{2}$ = 0.149]. Thus, the temporal weights in a given band were not strongly affected by the presence or absence of gap in the other band. However, as discussed in the introduction, there are both arguments for expecting frequency-specific as well as for expecting frequency-unspecific temporal weights. For this reason, we conducted separate Bayesian rmANOVAs that quantify the relative evidence for both variants for the weights displayed in each panel of Figure 3, using the software JASP (JASP Team, 2019). These analyses encompass all potential effects that might occur if the weights were somehow dependent between bands, as the analysis looks for any effect of context on the pattern of weights. In these analyses, we focused on the segments within and adjacent to the region of the gap as possible differences in the weighting patterns that would allow to differentiate between the two hypothesis were most likely to happen there. For each of these Bayesian rmANOVAs, the within-subjects factors were segment number (5–11 for bands without a gap, 5–6 for bands that contained a gap) and context (other band without a gap, other band with 500-ms gap). To quantify the influence of spectral context on the segment weights, we compared the posterior likelihood of the complete model that contained both main factors (segment number and context) and their interaction (segment number × context), to the posterior likelihood of a reduced model that included only the main factors segment number and context. The reduced model assumes no effect of spectral context on the temporal weights (that is, no segment × context interaction; *H*_0_), while the full model assumes that the spectral context affects the weights (segment × context interaction; *H*_1_). The scale parameter of the Cauchy prior distribution was set to commonly used values, i.e., *r* = 0.5 for fixed effects and *r* = 1.0 for random effects (for details on multivariate priors for Bayes factors see Rouder et al., 2012). We computed Bayes factors defined as the ratio between the posterior probability that the data occurred under *H*_0_ (model without the segment × context interaction) and the posterior probability that the data occurred under *H*_1_ (model including the segment × context interaction). Values of this Bayes factor (termed BF_01_ in the following) greater than 1.0 represent evidence in favor of the reduced model. For all four Bayesian rmANOVAs, the BF_01_ values were in favor of the reduced model not containing the interaction term, ranging from 2.08 (panel B) to 4.90 (panel C). This means that, for example, the patterns of weights displayed in panel B were 2.08 times more likely to occur under the null hypothesis of no segment × context interaction compared to the alternative hypothesis. According to the categories of Jeffreys (1961), there was thus anecdotal to moderate evidence for the null hypothesis that within each panel the segment weights within and around the region of the gap, depended only on the segments’ temporal position, but not on the context (i.e., on whether the other frequency band was presented with or without a gap). To assess the robustness of the Bayes factors, we changed the width of the prior distribution for fixed effects over a range from 0.15 to 1.5. The resulting BF_01_ values are plotted in Figure 4. The changes in prior width did not affect the direction of the stated results. However, the size of the factors showed substantial variation ranging from 1.22 (prior width *r* = 0.15, panel A) to 89.11 (prior width *r* = 1.5, panel C). Taken together, the direction of the results indicates that the weights for both bands were hardly affected by the presence or absence of a gap in the other band.

In summary, Experiment 1 provides two main findings. First, it confirms previous data showing a reoccurrence of the primacy effect on the second sound part of a frequency band when this band contained a gap (Fischenich et al., 2020). In conditions where a band contained a gap, the weight assigned to the first segment of that band following the gap was higher than the weight assigned to the same segment when the band did not contain a gap. Even more important for the present study, the second finding was that the weights assigned to a given frequency band were virtually unaffected by its spectral context – that is, by whether the other band did or did not contain a gap. This observation was supported by Bayesian rmANOVAs, which consistently showed Bayes factors in favor of a reduced model not containing the segment × context interaction. The results from Experiment 1 thus indicate that temporal weights in loudness judgments are frequency specific.

## Experiment 2

Experiment 1 showed a significant reoccurrence of the primacy effect for bands that contained a silent gap of 500-ms duration, and that the temporal weights were frequency specific. Experiment 2 was conducted to replicate these findings in an independent group of participants. Also, we presented a slightly longer gap duration of 700 ms compared to the 500 ms in Experiment 1. The reoccurrence of the primacy effect after a silent gap was reported to be more pronounced at longer gap durations (Fischenich et al., 2020). As a consequence, the presence or absence of the 700-ms gap was expected to cause a stronger change in the weights on the “context band” than for a 500-ms gap, and thus to provide a stronger test of our hypothesis that the temporal weights assigned to a given frequency band are independent of the temporal weights assigned to a remote frequency band. Apart from the longer gap duration, the stimuli, apparatus and procedure were identical to those used in Experiment 1.

### Method

#### Listeners

Eight normal hearing listeners (five female, three male, age 21–32 years) participated in this experiment. None of them had participated in Experiment 1. Hearing thresholds were measured by Békésy audiometry with pulsed 270-ms pure tones. All participants showed thresholds less than or equal to 15 dB HL bilaterally in the frequency range between 125 Hz and 8 kHz. All participants were students from the Johannes Gutenberg-Universität Mainz and received partial course credit for their participation.

#### Stimuli, Apparatus, Procedure, and Data Analysis

The apparatus was the same as used in Experiment 1. Except for the 700-ms gap duration, the stimuli were also identical to those presented in the previous experiment. The procedure and the data analysis were identical to Experiment 1. The average level difference between HB and LB at equal loudness was -3.27 dB (*SD* = 5.51 dB) and thus slightly larger than in Experiment 1. The individual mean sound pressure level differences between HB and LB at equal loudness are shown in Supplementary Table 2. As in Experiment 1, the loudness matches were stable across time (see Supplementary Material “Loudness matching” for more Information).

Table 3 shows the percentages of “softer” and “louder” responses as well as the SDT decision criterion c and the sensitivity in terms of d′ for each listener in Experiment 2.

**TABLE 3:** Average percentages of “softer” and “louder” responses as well as the SDT decision criterion *c* and the sensitivity in terms of *d*′ for each listener in Experiment 2.

Listener	% “louder”	% “softer”	Mean of *c*	SD of *c*	Mean of *d*′	SD of *d*′
1	0.59	0.41	–0.28	0.19	1.15	0.17
2	0.47	0.53	0.10	0.27	1.02	0.28
3	0.51	0.49	–0.03	0.16	0.71	0.28
4	0.51	0.49	–0.04	0.11	1.02	0.25
5	0.40	0.60	0.31	0.18	1.15	0.21
6	0.59	0.41	–0.24	0.18	0.52	0.23
7	0.63	0.37	–0.36	0.20	0.76	0.24
8	0.61	0.39	–0.32	0.30	0.79	0.18

Across the 32 fitted logistic regression models for all combinations of condition and listeners (eight listeners, four conditions), the area under the ROC curve ranged between 0.65 and 0.84 (*M* = 0.77, *SD* = 0.05), and was thus comparable to the values in Experiment 1.

### Results

We report the same analyses as in Experiment 1. The average sensitivity in terms of *d*′ is shown in Table 4 for each of the four conditions of Experiment 2. There was a significant effect of condition on *d*′, *F*(3,21) = 6.09, $\tilde{\varepsilon }$ = 0.658, *p* = 0.013, $\eta_{p}^{2}$ = 0.465, with slightly higher mean sensitivity when both bands contained a gap (condition LB_700_HB_700_).

**TABLE 4:** Mean sensitivity (*d*′) in the four different conditions of Experiment 2.

Condition	Mean of *d*′	SD of *d*′
LB_0_HB_0_	0.87	0.24
LB_0_HB_700_	0.85	0.27
LB_700_HB_0_	0.88	0.2
LB_700_HB_700_	1.00	0.23

**N* = 8.*

**FIGURE 5:**
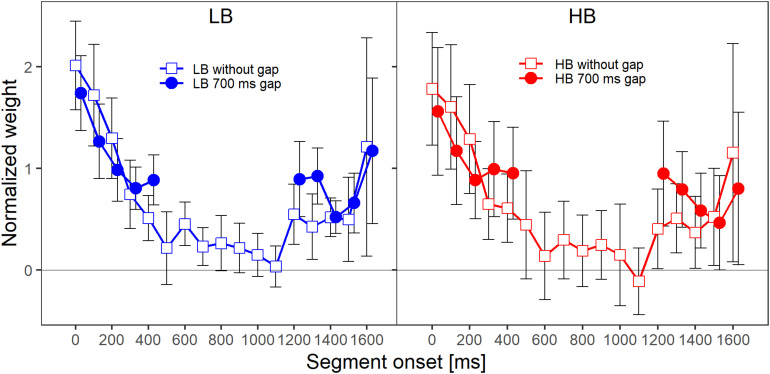
Mean normalized weights as a function of segment onset for Experiment 2, averaged across spectral context. The two panels show the weights for the two frequency bands, LB (lower band, **left panel**) and HB (higher band, **right panel**). Frequency band is also indicated by color (LB: blue, HB: red). The different symbols and separate lines within each panel indicate whether the band did or did not contain a gap (open squares: without gap, filled circles: 700-ms gap). Error bars show 95% confidence intervals (CIs). Note that for better visibility, the two lines are shifted slightly against each other along the *x*-axis.

Figure 5 shows the mean normalized temporal weights assigned to the two frequency bands. Filled circles and open squares represent conditions where the plotted band did or did not contain a gap, respectively. For each of the plotted lines, the weights are averaged across the spectral context, that is, across the two conditions where the other frequency band either did or did not contain a gap.

As in Experiment 1, for both frequency bands, the patterns of the mean weights in both conditions (with and without a gap) showed a clear primacy effect at the beginning of the sound, in the sense that the weight on the first segment was higher than the weights on the following segments. Furthermore, when a band contained a gap, the weight assigned to the first segment after the gap was higher compared to the condition in which the band did not contain a gap. An rmANOVA with the within-subjects factors segment number (1–5 and 13–17 for bands without a gap, 1–10 for bands that contained a gap), frequency band (lower, higher), target gap (no gap, 700-ms gap) and spectral context (no gap in other band, 700-ms gap in other band) was conducted. The main effect of segment number was not significant, *F*(9,63) = 2.98, $\tilde{\varepsilon }$ = 0.177, *p* = 0.10, $\eta_{p}^{2}$ = 0.298. This was likely caused by the response pattern of two listeners, who showed an almost exclusive weight on the last segment in almost all conditions for both bands (i.e., a strong recency effect; Oberfeld et al., 2018), while all remaining listeners showed a clear primacy effect. When these two listeners with strong recency effects were removed from the data analysis, the main effect of segment number was significant and comparable to the effect observed in Experiment 1, *F*(9,45) = 34.26, $\tilde{\varepsilon }$ = 0.524, *p* < 0.001, $\eta_{p}^{2}$ = 0.873.

For the rmANOVA including the data from all participants, there was a significant segment number × target gap interaction, *F*(9,63) = 3.25, $\tilde{\varepsilon }$ = 0.775, *p* = 0.007, $\eta_{p}^{2}$ = 0.317, indicating that the pattern of temporal weights differed depending on whether a band contained a gap or was presented without a gap. Thus, as in Experiment 1, we observed a significant reoccurrence of the primacy effect.

**FIGURE 6:**
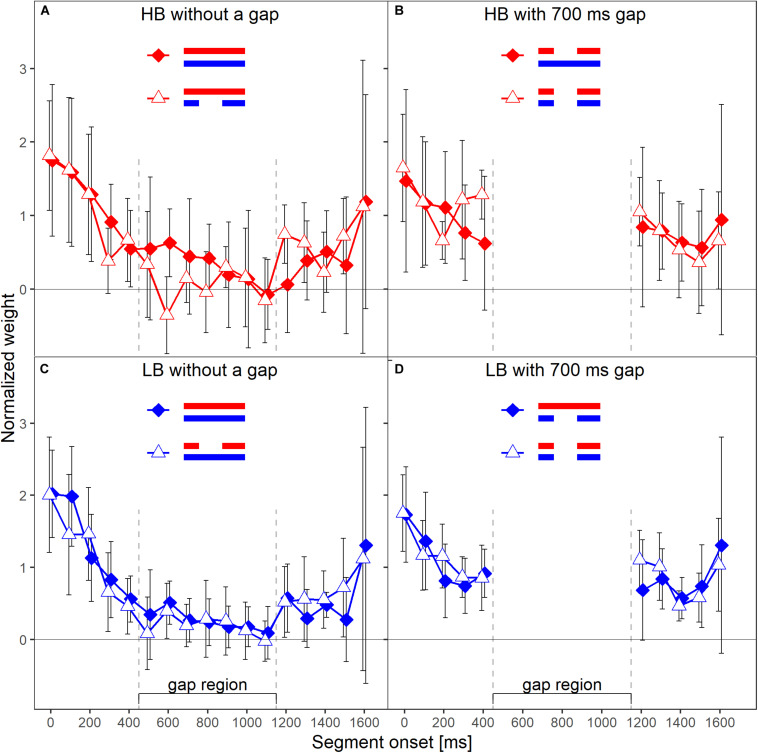
Mean normalized weights as a function of segment onset for Experiment 2. Upper panels **(A,B)** show the weights for the higher frequency band HB, lower panels **(C,D)** show the weights for the lower band LB. The frequency band is also indicated by symbol and line color, red = HB, blue = LB. Panels in the left column show the weights in the conditions without a gap in the plotted band, panels on the right show the weights in the conditions with a gap. In each panel, the two different lines indicate the two different context conditions. Solid diamonds show the weights in the conditions in which the other band did not contain a gap, open triangles show the weights in the conditions in which the other band contained a gap. Error bars show 95% confidence intervals (CIs). Note that for better visibility, the two lines are shifted slightly against each other along the *x*-axis.

Each panel in Figure 6 shows the normalized weights for one band and depending on whether or not the other band contained a silent gap. As in Experiment 1, to investigate the frequency-specificity of the weights, one has to compare the two lines in each panel of Figure 6 that represent the two spectral context conditions (other band presented with our without a gap). For the lower band (lower panels), the two patterns of weights displayed in each panel are very similar. Thus, the weights assigned to the lower band were virtually unaffected by the presence or absence of a gap in the other band for both HB and LB. For the higher band (upper panels), the weights obtained in the two different context conditions showed differences for a few segments. However, for most segments, the weights were similar across the two context conditions.

**FIGURE 7:**
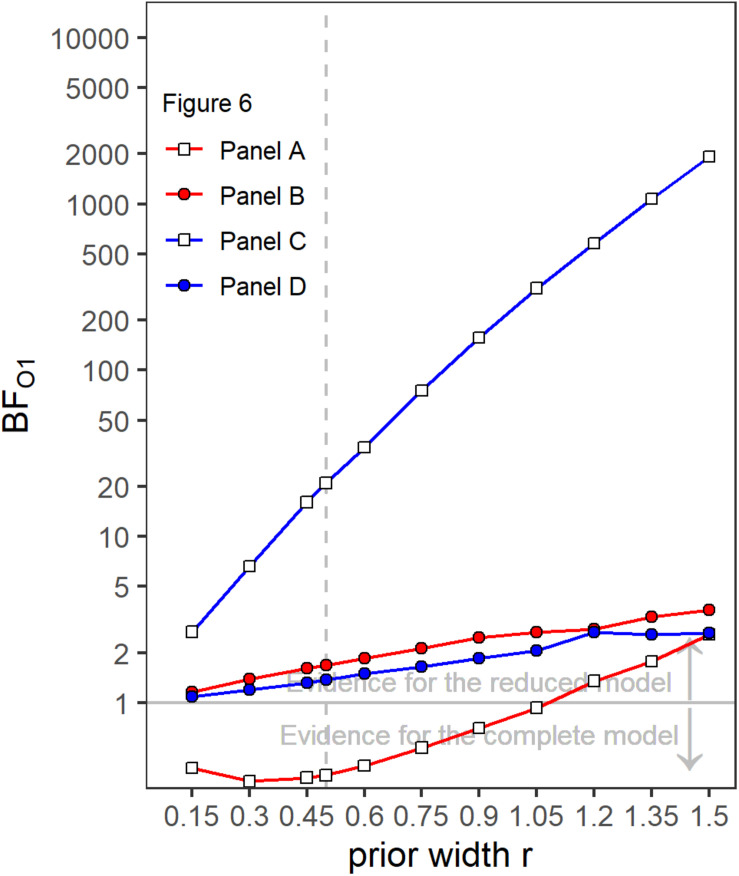
Bayes factors (BF_01_) as a function of prior width for the four different panels of Figure 6. Values of BF_01_ greater than 1.0 indicate a higher posterior probability for the reduced model not containing a segment × context interaction, compared to the complete model including a segment × context interaction.

Like in Experiment 1, the rmANOVA did not show significant interactions of the factor context with segment number [*F*(9,63) = 1.81, $\tilde{\varepsilon }$ = 1, *p* = 0.084, $\eta_{p}^{2}$ = 0.206], segment number and target gap [*F*(9,63) = 1.16, $\tilde{\varepsilon }$ = 0.685, *p* = 0.343, $\eta_{p}^{2}$ = 0.143] or segment number, target gap and target frequency band [*F*(9,63) = 1.13, $\tilde{\varepsilon }$ = 1, *p* = 0.297, $\eta_{p}^{2}$ = 0.139). Separate Bayesian rmANOVAs were conducted per panel (that is, per combination of target band and target gap conditions) with the within-subjects factor segment number (5–13 for bands without a gap, 5 and 6 for bands that contained a gap) and context (other band without a gap, other band with 700-ms gap). The complete model that contained both main factors (segment number and context) and their interaction (segment number × context) was compared to a reduced model that included only the main factors segment number and context. Three of the resulting BF_01_ values were in favor of the reduced model, ranging from 1.37 (panel D) to 20.95 (panel C). Only the BF_01_ value of 0.37 for panel A was in favor of the complete model, showing according to Jeffreys (1961) categories anecdotal evidence for an effect of context. The robustness of the Bayes factors to changes in prior width is shown in Figure 7. Changes in prior width did only affect the direction of the stated results for panel A, where with increasing prior width, the results were also in favor of the reduced model. For panel C, the size of the factors showed substantial variation ranging from 2.67 to 1923.95. In general, the results thus indicate that the weights for both bands were hardly affected by the presence or absence of a gap in the other band.

Thus, both of the main findings from Experiment 1 were confirmed in a different group of listeners and presenting a longer gap duration. There was a significant reoccurrence of the primacy effect on the second sound part when a frequency band contained a gap. For the majority of the analyses the weights assigned to a given band were largely unaffected by its spectral context, that is, by whether or not the other band contained a gap. The pattern of results thus confirms the conclusion from Experiment 1 that the temporal weights in loudness judgments are frequency specific.

## Discussion

The present study examined whether the temporal weights assigned to different frequency bands when listeners judge the overall loudness of a time-varying sound are frequency specific. In two experiments conducted in independent groups of listeners, temporal loudness weights were measured for stimuli consisting of two frequency bands. We introduced silent gaps in neither, only one, or both bands. According to previous research (Fischenich et al., 2020), silent gaps result in a reoccurrence of the primacy effect after the silent gap. The temporal weights for conditions where only one of the bands contained a silent gap were compared to the weights observed when both bands were contiguous (no gap) or when both bands contained a gap. For all conditions in both experiments, primacy effects at the onset of the sounds were observed, in the sense that the first segments of a sound received higher weights compared to the following segments. This is compatible with previous data (e.g., Pedersen and Ellermeier, 2008; Rennies and Verhey, 2009; Fischenich et al., 2019).

In Experiment 2, two listeners consistently showed strong recency effects rather than a primacy effect for both bands and in all gap conditions. In previous studies, recency effects appeared from time to time in some conditions and for some listeners (condition without feedback, Pedersen and Ellermeier, 2008; in Experiment 3 and 4, Oberfeld and Plank, 2011; for five segment sounds with durations of 2.5 s and above, Oberfeld et al., 2018; sounds in background noise SL 7.5 dB, Fischenich et al., 2019). In general, they are less frequent and less pronounced than the primacy effect. The primacy effect has been observed very consistently across a large number of studies (for a review see Oberfeld et al., 2018). However, inter-individual differences in perceptual weights tend to be rather large, showing various kinds of patterns (e.g., Lutfi et al., 2011). This is even more pronounced for recency effects (Oberfeld and Plank, 2011; Oberfeld et al., 2018).

Bands that contained a gap showed higher weights on the first segments following the gap, compared to the weights assigned to segments at the same temporal position when the band did not contain a gap. This difference in the weighting patterns was statistically significant in both experiments. Thus, the results confirm the finding that the primacy effect reoccurs after a silent gap of a certain duration within a sound (Fischenich et al., 2020).

The main aim of the present study was to answer the question of whether the temporal loudness weights are applied independently for each frequency band contained in the stimulus, or to both bands simultaneously. Across the two experiments, the general patterns of the temporal weights assigned to the target band were hardly affected by the spectral context (i.e., presence or absence of a silent gap in the other frequency band). However, descriptively the weights in the gap region were sometimes smaller when the other band contained a gap compared to when it did not contain a gap (see Figures 3A, 6A), indicating a context effect. If suppression of the HB by the LB and a resulting increase in loudness of the HB during the gap in the LB had played a role, the opposite pattern – higher weights on the HB weights during the gap region when the LB contained a gap – should have resulted. A potential explanation for these descriptive trends could rather be that loudness dominance takes place *after* spectral integration and therefore parts of the sound where both bands were present received higher weights. However, under this assumption, one should expect the weights in the continuous band to show a much stronger decline when the other band contains a gap. A reduction in sound pressure level by 10 dB has been shown to result in almost zero weights (e.g., Oberfeld and Plank, 2011). Because the two bands were loudness-matched in our experiments, we can assume that the total loudness during the gap in one band was approximately half of the total loudness when both bands were present. Thus, the effect of the gap on total loudness can be expected to be similar to the effect of a 10-dB level reduction within a single band, which also corresponds to a loudness reduction by approximately a factor of two. In addition, the loudness dominance effect would also have resulted in greater differences between all of the weights after the gap compared to the weights within the gap (see Figure 5 in Fischenich et al., 2020). In addition, in Figure 3C, the weight on the first segment of the LB within the gap region in the HB was higher (rather than lower) when the other band did contain a gap, compared to when it did not contain a gap. This illustrates the variability in the data, as some descriptive data were compatible with an effect of spectral context, but the data also showed descriptive weight differences comparable in size that are incompatible with the assumption.

Another example for a descriptive pattern in the data that could be taken as an effect of spectral context is that in the continuous HB (without gap), the weight difference between the last segment within the gap region of the other band and the first segment after the gap region of the other band was higher when the other band contained a gap, compared to when the other band did not contain a gap. Interestingly, this pattern was present only for the continuous HB, but not for the continuous LB, in both experiments (see Figures 3A,C, 6A,C). If one assumes that the gap in the other band caused an additional onset effect also in the ongoing band, it is difficult to argue why this was the case only for the HB, but not for the LB. Also, if one assumes higher loudness of the HB during the gap in the LB due to suppression, the first HB segment following the gap region should have been perceived as softer than the last HB segment in the gap region, due to suppression by the LB that was again present for this segment. In such a case, it is difficult to understand why then the weight on the first HB segment following the gap region was *higher* rather than lower when the LB contained a gap.

Apart from these relatively small effects of spectral context in a small subset of the weights, the more systematic and encompassing statistical evaluation of the size of the context effect, which was provided by the Bayes factors (BF_01_) that compared the posterior likelihood for a model with an effect of context (i.e., assuming that the gap in the other band has a systematic effect on the weights for the target band), and a model without this effect of context, showed evidence for an absence of an effect of spectral context on the temporal weights for a given frequency band, for most of the conditions. The results thus indicate that the temporal weights in loudness judgments are, by and large, frequency specific.

In the context of loudness models, this finding suggests a weighting on the basis of a time-varying specific loudness, i.e., the loudness time function at the output of each frequency channel (auditory filter). The debate on whether temporal integration precedes spectral integration was already present when Zwicker (1977) proposed his original loudness model for time-varying sounds. Zwicker argued on the basis of results indicating spectral loudness summation for non-simultaneously presented frequency components (Zwicker, 1969; see also Heeren et al., 2011) that spectral integration should precede temporal integration. Thus, the dynamic loudness model (DLM) (Zwicker, 1977) and models based on it (Chalupper and Fastl, 2002) assume that spectral integration precedes temporal integration. The same order of the processing stages was assumed in the time-varying loudness model (TVL-model) proposed by Glasberg and Moore (2002). However, in the most recent versions of this model (Moore et al., 2016, 2018), the short-term specific loudness is calculated per frequency channel before spectral summation takes place. The assumption that temporal processing precedes spectral integration is compatible with the present finding of frequency-specific temporal loudness weights. It is also compatible with neurophysiological data showing entrainment to channel-specific instantaneous loudness in cortical MEG components up to about 100 ms (Thwaites et al., 2017). One should keep in mind, however, that the attack-decay type of temporal integration assumed by the TVL-model does not predict a primacy effect, as demonstrated by simulation results in Fischenich et al. (2019).

A possible explanation for the observed reoccurrence of the primacy effect within a frequency band when the band contained a gap might be that the re-onset of the band containing the gap might in principle capture the attention (Oberfeld and Plank, 2011). Such an attentional capture could cause a primacy effect on the post-gap part of the band containing the gap (if the weights are assigned per band), and even also on the band that did not contain a gap (if the weights are assigned across frequency). However, our previous work did not provide compelling support for such an attention-orienting explanation of the primacy effect. A reduction of the perceived abruptness of the onset effect by presenting the target sound in continuous background noise (Fischenich et al., 2019), or by imposing a gradual fade-in in level at the sound onset (Oberfeld and Plank, 2011), did not remove the primacy effect pattern.

Fischenich et al. (2020) discussed three possible explanations for the primacy effect and its reoccurrence after a silent gap. The first explanation, originally proposed by Oberfeld and Plank (2011), is based on the response characteristics of neurons in the AN, which tend to show a peak in the firing rate at the sound onset (Nomoto et al., 1964; Rhode and Smith, 1985). The inter-stimulus-interval that was reported to be necessary to see a reoccurrence of the initial peak in the firing rate of some types of nerve fibers (Relkin and Doucet, 1991) is roughly in line with the necessary interval to see a significant reoccurrence of the primacy effect (Fischenich et al., 2020). Because the inner hair cells that innervate the AN fibers are frequency specific, the recovery of the firing rate is also frequency specific (Harris and Dallos, 1979). The explanation of the primacy effect in temporal loudness weights based on the response characteristics of the AN fibers is thus compatible with the result of frequency-specific weights in the present study. However, the inter-individual differences in weighting patterns with pronounced recency effects for two listeners in Experiment 2 argue against an explanation based on the response characteristic of the AN. If the weighting patterns were due to the initial peak in the firing rate of the AN fibers, cases in which individuals show a completely reversed weighting pattern with strong recency effects should not occur.

A second potential explanation of the primacy effect and its reoccurrence is based on research on masking effects on intensity discrimination, which shows that for masker-target intervals below 400 ms, intensity-difference-limens (DLs) are increased substantially (e.g., Zeng et al., 1991; Oberfeld, 2008b). A segment presented in the middle of a longer sound might be subject to forward masking by preceding segments, which would result in a primacy effect if listeners adopted a reasonable strategy of placing higher weights on temporal portions of a sound for which the intensity resolution is higher (Green, 1958; Oberfeld et al., 2013). The silent gap necessary for a significant reoccurrence of the primacy effect in Fischenich et al. (2020) was approximately in line with the time course of masking effects on DLs. The explanation of the primacy effects and its reoccurrence based on masking effects on intensity discrimination, are in line with frequency-specific weights, because no DL elevations were observed when the masker-signal frequency separation is large (Zeng and Turner, 1992). However, as discussed in detail in Fischenich et al. (2020), several additional assumptions are needed in order to explain the primacy effect in temporal loudness weights by masking effects on intensity resolution.

A third potential explanation of the primacy effect and its reoccurrence is provided by an *evidence integration approach* (e.g., Vickers, 1970). Evidence integration suggests that when making perceptual judgments, listeners accumulate evidence for each of the possible response alternatives in a random walk process. As discussed by Fischenich et al. (2020), models that simulate such an evidence accumulation process can produce temporal weighting patterns with either primacy or recency effects. If one assumes that a separate evidence integration process is in effect for each frequency band or auditory stream, then frequency-specific weights are predicted. Furthermore, if one assumes that after a gap of sufficient duration within a band, a separate evidence integration process is carried out for both temporal parts of the band (the part before and the part after the gap), then evidence integration can also account for the reoccurrence of the primacy effect within a band.

It should be noted that while all three of the potential explanations of the frequency specificity of the temporal weighting patterns account for some aspects of the observed results, each of them has some clear limitations (for a discussion see Fischenich et al., 2020). It is currently not possible to decide which of the alternative mechanisms is the most likely explanation of the observed temporal loudness weights.

In addition, in an absolute identification task as the one presented in the experiments of this study, the decision of a participant might depend not only on the segment levels presented on the current trial, but also on the sounds presented on preceding trials (e.g., Stewart et al., 2005). It would be interesting to investigate such potential sequential effects in future research.

To summarize, in two experiments, the present study investigated whether the temporal weights assigned to different frequency bands when listeners judge the overall loudness of a time-varying sound are frequency specific. The results of both experiments indicated that temporal loudness weights are approximately frequency specific. While the frequency specificity of the weights is in accordance with several potential explanations of the primacy effect in loudness judgments, further research is needed to investigate the underlying mechanisms of the primacy effect as well as of its recovery during silent gaps.

## Data Availability Statement

The datasets presented in this study can be found in online repositories. The names of the repository/repositories and accession number(s) can be found below: https://osf.io/qh3zt.

## Ethics Statement

The studies involving human participants were reviewed and approved by Ethics Committee of the Institute of Psychology of the Johannes Gutenberg-Universität Mainz (reference number 2016-JGU-psychEK-002). The patients/participants provided their written informed consent to participate in this study.

## Author Contributions

AF and DO supervised the data acquisition and data curation, performed the statistical analyses, and created the graphical illustrations and tables. DO and JV administered and supervised the project. DO was responsible for the funding acquisition. All the authors participated in writing the original draft and contributed to the development of the method and software, manuscript revision, and read and approved the submitted version.

## Conflict of Interest

The authors declare that the research was conducted in the absence of any commercial or financial relationships that could be construed as a potential conflict of interest.

## Supplementary Material

Supplementary Data Sheet 1 Supplementary Material Loudness matching.

Supplementary Data Sheet 2 Supplementary Material Alternative Normalization.
