# Supplementary material for: Temporal Loudness Weights Are Frequency Specific
Source: Front Psychol. 2021 Mar 19;12:588571. doi: 10.3389/fpsyg.2021.588571 (PMC8017310; doi:10.3389/fpsyg.2021.588571)
Supplement: Supplementary Data Sheet 1 — Supplementary Material Loudness matching. [file Data_Sheet_1.pdf]

Supplementary Material for the paper "Temporal loudness weights are frequency specific", 2021, Frontiers in Psychology - Auditory Cognitive Neuroscience, doi: 10.3389/fpsyg.2021.588571

Alexander Fischenich[1*], Jan Hots[2], Jesko Verhey[2], and Daniel Oberfeld[1]

[1] Department of Psychology, Johannes Gutenberg-Universität Mainz, Mainz, Germany

[2] Department of Experimental Audiology, Otto von Guericke University Magdeburg, Magdeburg, Germany

* Corresponding author

E-mail: alexander.fischenich@uni-mainz.de, oberfeld@uni-mainz.de

LOUDNESS MATCHING

For each listener, loudness matches between the low and the high noise band (LB and HB) were obtained in the first session of the experiment with an adaptive two-interval forced-choice task, prior to the main loudness judgment task. On each trial, listeners compared the loudness of one segment of the HB to the loudness of one segment of the LB. The duration of each segment was 120 ms including 20-ms on- and off ramps, and thus identical to the segment duration in the main loudness judgment task. The inter stimulus interval between the two sounds was 650 ms. The adaptive procedure varied the sound level of either the LB or the HB. Thus, the comparison stimulus (level varied by the adaptive procedure) was either LB or HB, while the other band (the standard) was fixed in level. In addition, we varied the presentation order, so that the comparison was presented either in the first or in the second interval. The comparison stimulus (LB or HB) and the presentation order (comparison presented first or second) were combined factorially, resulting in four adaptive tracks. To further reduce biases, these four adaptive tracks were randomly interleaved within an experimental block (cf. Buus et al., 1997; Verhey, 1999; Oberfeld et al., 2012). On each trial, the participant indicated which of the two sounds had been louder by pressing the

corresponding button on a numeric keypad. At the beginning of each adaptive track, both the standard and the comparison were presented with a sound pressure level of 52.0 dB SPL, corresponding to the grand mean of the level distributions for the LB in the main loudness judgment task. The sound pressure level of the comparison was changed according to an adaptive 1-up, 1-down rule (Levitt, 1971) with an initial step size of 5 dB for the first four reversals and a step size of 2 dB for the remaining reversals of the adaptive track. A reversal was defined as a trial on which the response ("first louder" or "second louder") differed from the response on the previous trial of this particular adaptive track (i.e., "peaks" and "valleys" in the adaptive track; Levitt, 1971). For each of the four tracks, a minimum of 15 reversals was obtained. Trials from adaptive tracks for which 15 reversals had already occurred continued to be presented with an a-priori probability of 0.125, to prevent that, at the end of a block, only one or two conditions are presented. For each track, the first four reversals (with the large step size) were excluded from the data analysis. The arithmetic mean of the comparison levels at the maximally possible even number of remaining reversals was used to calculate the level difference between the comparison and the equally loud standard, that is, the loudness match. The resulting sound pressure level differences between HB and LB at equal loudness were averaged across the four tracks. After a practice block containing 30 trials (not included in the data analysis), two blocks of the matching task were presented, each comprising the four randomly interleaved tracks described above. For each listener, the arithmetic mean of the average loudness matches in the two blocks was calculated. Adaptive tracks for which the standard deviation of the comparison level at the counting reversals was higher than 5 dB were excluded from the calculation.

Table1 shows the resulting individual mean sound pressure level differences between HB and LB at equal loudness ($\Delta L_{\mathrm{H-L}}$) obtained in the initial session of Experiment 1. In the last session of the experiment, the loudness matching procedure of all stimulus pairs was repeated, to investigate whether the loudness matches were stable across time. The individual

loudness matches obtained in the final session of Experiment 1 are also displayed in Table 1. The average ΔL_{H-L} obtained in session 1 (blocks 1 and 2, $M = -0.273$ dB, $SD = 4.11$ dB) did not differ significantly from ΔL_{H-L} in the final session (block 3, $M = -0.247$, $SD = 4.76$ dB), $t(7) = 0.021$, $p = .984$. For all but two listeners, the absolute value of the difference between the loudness matches in the first and final session was smaller than 3 dB. Intra-class correlation coefficients (ICC) and their 95% confidence intervals were calculated based on a mean-rating ($k = 2$), absolute-agreement, 2-way mixed-effects model (McGraw and Wong, 1996). The average ICC(A,2) of .828 with a 95% CI of .045 - .97 points to, on average, good agreement between the loudness matches in the first and in the final session.

Table 1: Loudness-matches in Experiment 1. Individual mean sound pressure level differences between the high and the low frequency band (ΔL_{H-L}) at equal loudness (and the corresponding standard deviation of ΔL_{H-L} across the adaptive tracks) obtained in the first and the final session. These individual matches were used to present the two bands at equal loudness in the main loudness-judgment task.

Listener	Session 1		Final Session	
	Mean ΔL_{H-L} (dB)	*SD* of ΔL_{H-L}	*Mean* ΔL_{H-L} (dB)	*SD* of ΔL_{H-L}
1	−3.79	2.97	−7.36	2.39
2	0.35	2.65	0.16	1.23
3	−0.58	1.57	−3.07	2.22
4	0.99	1.74	3.30	1.00
5	2.57	1.80	−0.02	0.52
6	6.68	1.61	8.50	3.07
7	−1.30	0.70	−3.48	0.94
8	−7.09	2.63	−0.01	1.11

Table 2 shows the resulting individual mean sound pressure level differences between HB and LB ($\Delta L_{H–L}$) at equal loudness obtained in the initial session of Experiment 2. In the last session of the experiment, the loudness matching procedure of all stimulus pairs was repeated, to investigate whether the loudness matches were stable across time. The individual loudness matches obtained in the final session of Experiment 2 are also displayed in Table 2. $_{H–L}$ The average $\Delta L_{H–L}$ obtained in session 1 (blocks 1 and 2, $M = −3.27$ dB, $SD = 5.51$ dB) did not differ significantly from $\Delta L_{H–L}$ in the final session (block 3, $M = −2.39$ dB, $SD = 3.46$ dB), $t(7) = -1.379$, $p = .210$. For all but two listeners, the absolute value of the difference between the loudness matches in the first and final session was smaller than 3 dB. The average ICC(A,2) of .883 with a 95%CI of .473 - .976 points to on average good agreement between the two measures.

Table 2: Loudness-matches in Experiment 2. Individual mean sound pressure level differences between the high and the low frequency band ($\Delta L_{H–L}$) at equal loudness (and the corresponding standard deviation across the adaptive tracks) obtained in the first and the final session.

Subject	$_{H–LH–L}$Session 1		Final Session	
	Mean $\Delta L_{H–L}$	*SD* of $\Delta L_{H–L}$	*Mean* $\Delta L_{H–L}$	*SD* of $\Delta L_{H–L}$
1	−6.50	3.29	−3.52	1.09
2	−7.43	3.03	−6.43	2.13
3	−5.26	2.01	−2.19	1.96
4	1.60	3.71	0.46	3.78
5	−5.24	3.33	−4.73	3.09
6	−1.51	1.30	−1.08	1.31
7	5.21	1.93	2.76	0.52

8	−10.13	5.61	−4.42	1.86

References

Buus, S., Florentine, M., and Poulsen, T. (1997). Temporal integration of loudness, loudness discrimination and the form of the loudness function. *Journal of the Acoustical Society of America* 101**,** 669-680.

Levitt, H. (1971). Transformed up-down methods in psychoacoustics. *Journal of the Acoustical Society of America* 49**,** Suppl 2:467-477.

McGraw, K.O., and Wong, S.P. (1996). Forming inferences about some intraclass correlation coefficients. *Psychological Methods* 1**,** 30-46.

Oberfeld, D., Heeren, W., Rennies, J., and Verhey, J. (2012). Spectro-temporal weighting of loudness. *PLOS One* 7**,** e50184.

Verhey, J. L. (1999). *Psychoacoustics of Spectro-Temporal Effects in Masking and Loudness Perception* Oldenburg: BIS-Verlag.
