# Supplementary material for: Temporal Loudness Weights Are Frequency Specific
Source: Front Psychol. 2021 Mar 19;12:588571. doi: 10.3389/fpsyg.2021.588571 (PMC8017310; doi:10.3389/fpsyg.2021.588571)
Supplement: Supplementary Data Sheet 2 — Supplementary Material Alternative Normalization. [file Data_Sheet_2.pdf]

Supplementary Material for the paper "Temporal loudness weights are frequency specific", 2021, Frontiers in Psychology - Auditory Cognitive Neuroscience, doi: 10.3389/fpsyg.2021.588571

Alexander Fischenich[1*], Jan Hots[2], Jesko Verhey[2], and Daniel Oberfeld[1]

[1] Department of Psychology, Johannes Gutenberg-Universität Mainz, Mainz, Germany

[2] Department of Experimental Audiology, Otto von Guericke University Magdeburg, Magdeburg, Germany

* Corresponding author

E-mail: alexander.fischenich@uni-mainz.de, oberfeld@uni-mainz.de

ALTERNATIVE NORMALIZATION

Following the suggestion of an anonymous reviewer, we conducted the entire data analyses of both experiments again, based on a different normalization of the weights. Here, the weights were normalized so that the mean of the absolute values of the weights on the first five segments was 1.0, for each combination of listener, target frequency band, target band gap, and context band gap.

For Experiment 1, each panel in Figure S1 shows the weights normalized in this manner for one band and depending on whether or not the plotted band did or did not contain a silent gap. If one compares this figure to the corresponding Figure 3 in the paper, it is evident that the weight patterns with the alternative normalization are very similar to the normalized weights analyzed in the manuscript (Figure 3 in the paper).

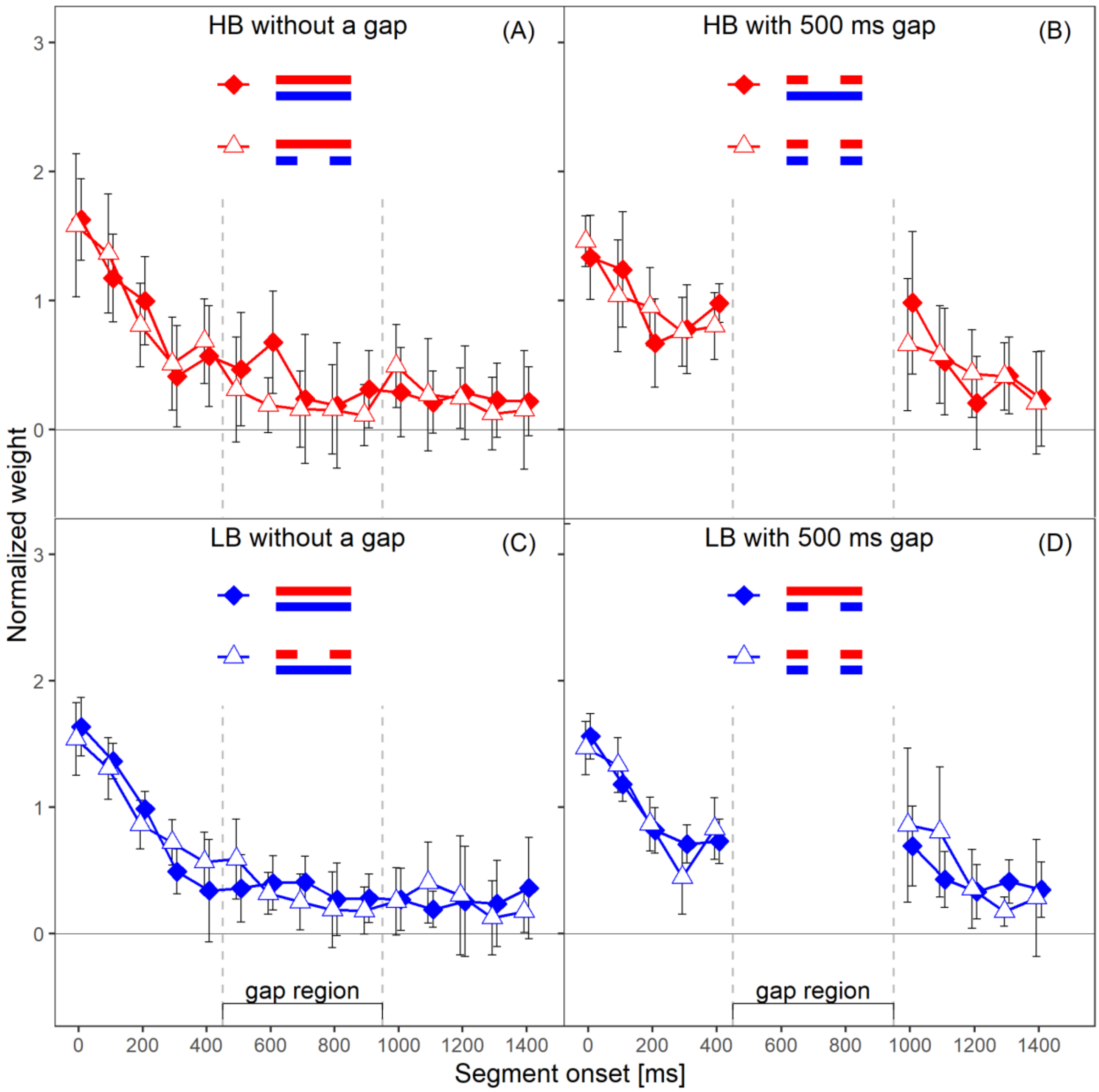

Figure S1: Mean normalized temporal weights as a function of segment onset for Experiment 1. The weights were normalized so that the mean of the absolute values of the weights on the first five segments was 1.0.. Upper panels show the weights for the HB, lower panels show the weights for the LB. The frequency band is also indicated by color, red = HB, blue = LB. Panels in the left column show the weights in the conditions without a gap in the analyzed band, panels on the right show the weights in the conditions with a gap in the analyzed band. In each panel, the two different lines indicate the two different context conditions. Solid diamonds show the weights in the conditions in which the other band did not contain a gap, open triangles show the weights in the conditions in which the other band contained a gap.

Error bars show 95% confidence intervals (CIs). Note that for better visibility, the two lines are shifted slightly against each other along the x-axis.

An rmANOVA showed a significant effect of segment number and a significant interaction target gap × segment number, but no significant effects involving context.

- Segment number , $F(9, 63) = 38.88$, $\tilde{\varepsilon} = .539$, $p < .001$, $\eta_p^2 = .847$,
- Target gap × segment number, $F(9, 63) = 3.56$, $\tilde{\varepsilon} = .774$, $p = .004$, $\eta_p^2 = .337$
- Context × segment number, $F(9, 63) = 1.21$, $\tilde{\varepsilon} = .960$, $p = .305$, $\eta_p^2 = .148$
- Context × segment number × target gap, $F(9, 63) = 1.64$, $\tilde{\varepsilon} = .993$, $p = .123$, $\eta_p^2 = .190$
- Context × segment number × target gap × target frequency band, $F(9, 63) = 1.40$, $\tilde{\varepsilon} = .995$, $p = .207$, $\eta_p^2 = .167$

These statistical results are very similar to the results of the corresponding rmANOVA for the normalization that was used in the main paper.

We also recalculated the Bayesian rmANOVAS per panel of Figure S1 (see Figure S2). The direction of evidence, always favouring the reduced model without the segment number × context interaction, is compatible with the analysis reported in the manuscript (see Figure 4 in the paper).

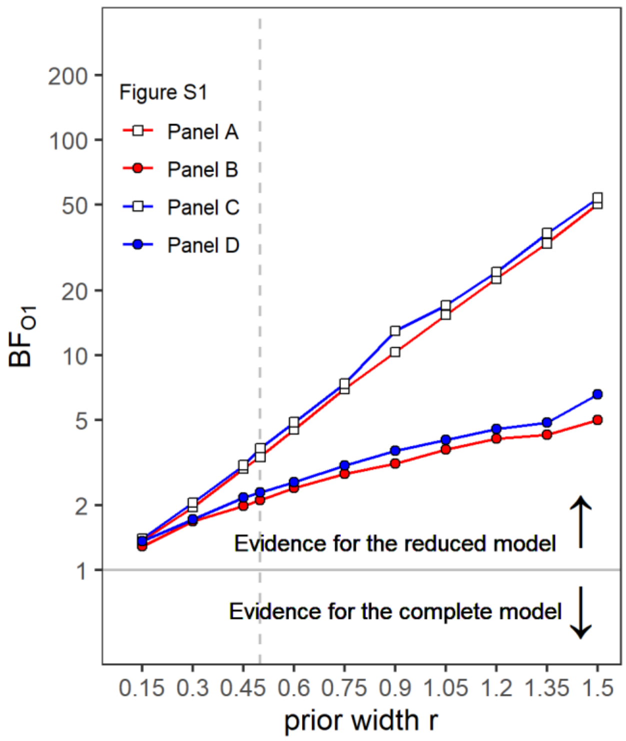

Figure S2: Bayes factors (BF01) as a function of prior width for the four different panels of Figure 1. Values of BF01 greater than 1.0 indicate a higher posterior probability for the reduced model not containing a segment × context interaction compared to the complete model including a segment × context interaction.

In Experiment 2, for one participant showing a pronounced recency effect (as described in the paper), slightly negative weights occurred on segments 1-5 in almost all conditions. This effect was most pronounced for the HB when it contained a gap and the LB did not contain a gap. In this condition, all of the first five segment weights for the respective listener were negative. For this reason, the weight normalization based on only the first five segments was not possible, and the participant was excluded from all of the following analyses. Figure S3 shows the weights from Experiment 2 normalized in the way stated above.

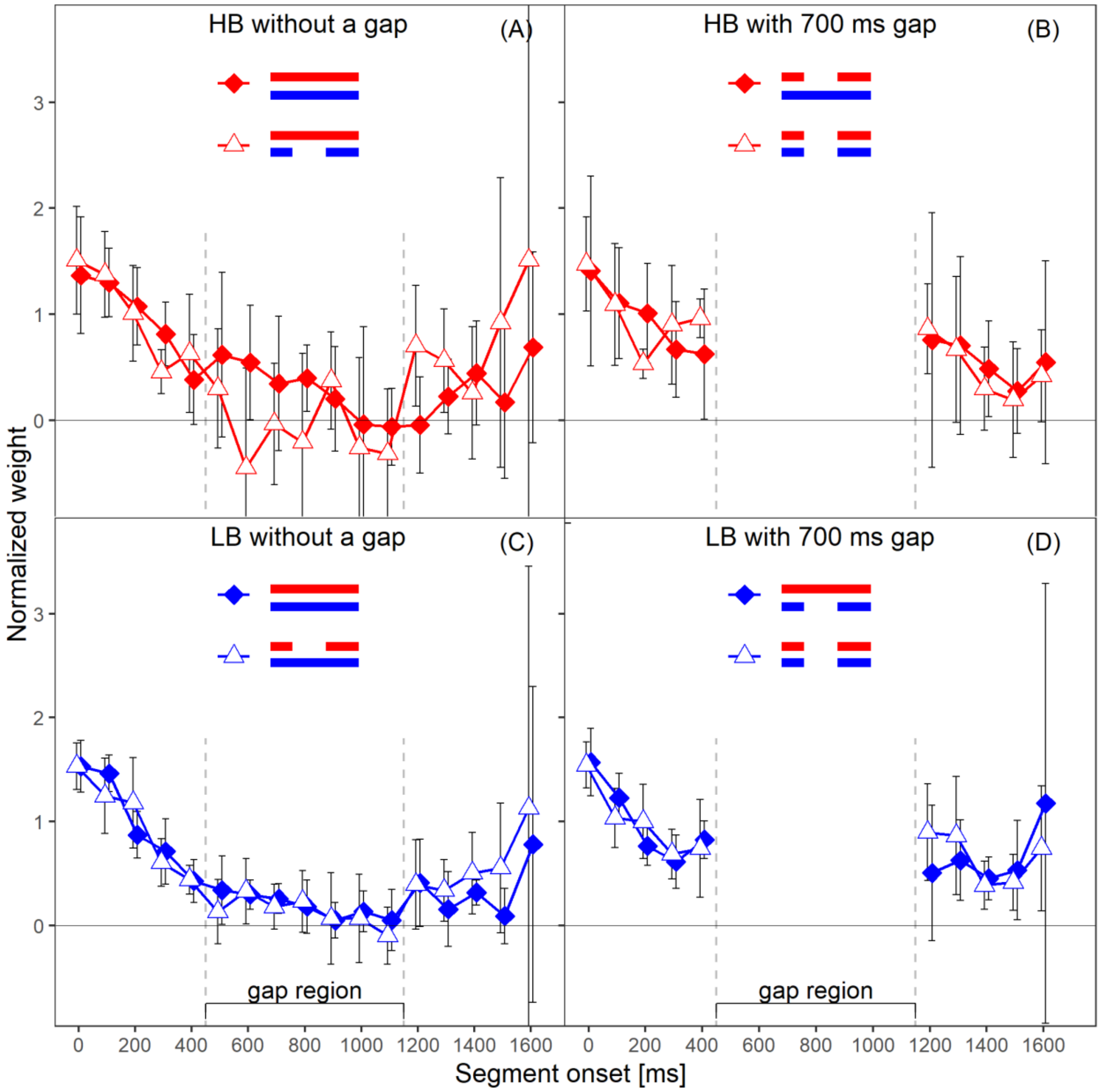

Figure S3: Mean normalized temporal weights as a function of segment onset for Experiment 2, when one listener was removed from the data because of an accumulation of negative weights on the first five segments. The weights were normalized so that the mean of the absolute values of the weights on the first five segments was 1.0. Upper panels show the weights for the HB, lower panels show the weights for the LB. The frequency band is also indicated by color, red = HB, blue = LB. Panels in the left column show the weights in the conditions without a gap in the analyzed band, panels on the right show the weights in the conditions with a gap in the analyzed band. In each panel, the two different lines indicate the two different context conditions. Solid diamonds show the weights in the conditions in which

the other band did not contain a gap, open triangles show the weights in the conditions in which the other band contained a gap. Error bars show 95% confidence intervals (CIs). Note that for better visibility, the two lines are shifted slightly against each other along the x-axis.

The pattern of results for Experiment 2 showed some small differences compared to the normalization presented within the manuscript (see Figure 6 in the paper). The interaction between segment number and target gap did not reach significance and the segment number × context interactions, which are most important for the present paper, showed even smaller effect sizes, and *p*-values that failed to reach significance clearly.

- Segment number , $F(9, 54) = 2.66$, $\tilde{\varepsilon} = .147$, $p = .139$, $\eta_p^2 = .294$,
- Target gap × segment number, $F(9,54) = 1.77$, $\tilde{\varepsilon} = .364$, $p = .183$, $\eta_p^2 = .228$
- Context × segment number, $F(9, 54) = 1.70$, $\tilde{\varepsilon} = .744$, $p = .141$, $\eta_p^2 = .220$
- Context × segment number × target gap, $F(9, 54) = 0.71$, $\tilde{\varepsilon} = .223$, $p = .509$, $\eta_p^2 = .106$
- Context × segment number × target gap × target frequency band, $F(9, 54) = 1.03$, $\tilde{\varepsilon} = .786$, $p = .428$, $\eta_p^2 = .146$

We also recalculated the Bayesian rmANOVAs for Experiment 2 (see Figure S4 below), which again showed a similar pattern as the analyses reported in the main paper (see Figure 7 in the paper).

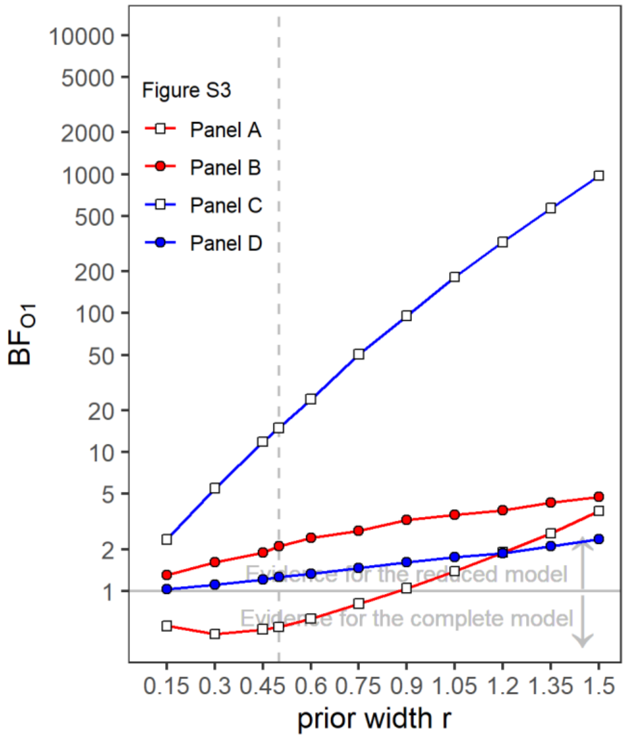

Figure S4: Bayes factors (BF01) as a function of prior width for the four different panels of Figure S3. Values of BF01 greater than 1.0 indicate a higher posterior probability for the reduced model not containing a segment × context interaction compared to the complete model including a segment × context interaction.

Taken together, the alternative weight normalization produces roughly the same pattern of results as the normalization used in the main paper. The normalization reported in the main paper also including the segments *after* the gap can be considered as more robust, because a higher number of weights is included in the normalization. For instance, the problems with negative weights on the first five segments only occurred for the alternative normalization reported in this supplementary analysis.
